# Age-adjusted visceral adiposity index (VAI) is superior to VAI for predicting mortality among US adults: an analysis of the NHANES 2011–2014

**DOI:** 10.1007/s40520-023-02660-z

**Published:** 2024-02-07

**Authors:** Wenwu Liu, Shuwei Weng, Yang Chen, Chenghui Cao, Daoquan Peng

**Affiliations:** Department of Cardiovascular Medicine, Research Institute of Blood Lipids and Atherosclerosis, the Second Xiangya Hospital of Central South University, No.139 Middle Renmin Road, Changsha, 410011 Hunan China

**Keywords:** Abdominal obesity, Aging, Visceral adiposity index, Cardiovascular diseases, National Health and Nutrition Examination Survey

## Abstract

**Background:**

The association of visceral adiposity with mortality in older adults is conflicting. Whether age influences the predicting ability of visceral adiposity (VAI) for mortality remains unknown. This study uncovered the relationship between age-adjusted visceral adiposity index and mortality through the data of NHANES 2011–2014.

**Methods:**

This study obtained data from the National Health and Nutrition Examination Survey (NHANES) 2011–2014. The age-adjusted visceral adiposity index (AVAI) scores were expressed as quartiles. Receiver operating characteristics (ROC) curve analysis was also applied to compare the predictive ability for mortality. Multivariate weighted Cox regression models were constructed to explore the association between AVAI and mortality. Kaplan–Meier survival curves were conducted for survival analyses. Smooth curve fittings and two-piecewise linear models were applied to explore the relationships between AVAI and mortality.

**Results:**

This study recruited 4281 subjects aged ≥ 18 years from the NHANES 2011–2014. The AUCs of AVAI were 0.82 (0.79, 0.86) and 0.89 (0.85, 0.92) for predicting all-cause mortality and cardiovascular mortality, which were superior to BMI, WC and VAI (all *p* < 0.05). AVAI is still an independent predictor for mortality adjusted for confounders. The associations of AVAI with all-cause and cardiovascular mortalities were dose-responsive, with higher AVAI scores indicating higher mortality risks.

**Conclusion:**

Age significantly improves the ability of VAI for predicting all-cause and cardiovascular mortality. Age-adjusted VAI is independently associated with mortality risk, and thus could be considered a reliable parameter for assessing mortality risk.

## Introduction

Obesity has been recognized as a worldwide epidemic problem leading to a heavy health burden for both individuals and society [1]. The deleterious influences of obesity, such as type 2 diabetes, cancer, and cardiovascular diseases (CVD), have been widely recognized. However, obesity, measured by the body mass index (BMI), has been demonstrated to be protective in older adults, which was termed as “the obesity paradox” [2]. The underlying mechanisms of this phenomenon are complex, but one of them is that BMI does not take the heterogeneity of body fat deposition into account. Mounting evidence showed that the distribution of excessive adiposity, but not obesity itself, impacts cardiovascular diseases and other chronic disorders [3–6]. It is important to consider body fat distribution when assessing the influence of obesity on health outcomes. Studies indicate that visceral adipose tissue (VAT) is a better parameter representing obesity than whole fat mass [7]. However, methods to measure VAT are expensive and time-consuming, e.g., computed tomography (CT) and magnetic resonance imaging (MRI). Waist circumference (WC) can better reflect the distribution of excessive adipose tissue than BMI [8], but it failed to differentiate subcutaneous and visceral fat well. Moreover, the function of visceral fat differs among individuals [9], making it necessary to develop a parameter that reflects the distribution and function of adipose tissue better and cost-effectively.

Visceral adiposity index (VAI) was created to assess the function of visceral adiposity. VAI is independently associated with cardiovascular and cerebrovascular events in Italians [10]. The sex-specific index comprises simple anthropometric measures and common lipidemic parameters. The VAI formula has BMI, WC, serum triglycerides (TG), and HDL cholesterol (HDL-C) and can reflect visceral adiposity dysfunction. Therefore, VAI is a more useful tool for predicting the incidence of diabetes than its components [11].

However, the association between VAT and all-cause mortality in elders remains conflicting according to previous studies [12–15]. Some studies showed that central obesity was associated with higher mortality risk [13–15], while other longitudinal studies demonstrated that greater VAT fails to predict higher all-cause mortality [12, 16]. The contradictions in these studies suggest that VAI may not be a comprehensive indicator of mortality risk. Age, perhaps an overlooked important factor, might be associated with these inconsistencies. Previous research has demonstrated a close relationship between age and changes in body composition, including reduced muscle mass, increased body fat, and fat infiltration in muscles, all of which are closely associated with functional decline and mortality in the geriatric population. Aging is also associated with changes in localized fat distribution and increased visceral fat mass [17–19]. One study has shown that age significantly improves the model fitting for visceral fat area and created a new index to reflect visceral fat volume and function, known as the Chinese Visceral Adiposity Index (CVAI).[20].Therefore, age is an important factor that cannot be ignored. Aging plays a crucial role in mortality and many chronic diseases, including cancer, neurodegeneration, and CVD. Thus, we seek to develop a new index including BMI, WC, HDL-C, TG, and age, termed age-adjusted VAI (AVAI), to better reflect the role of age in VAI. This study aimed to discover the association of AVAI and mortality, and further validate whether AVAI is superior for predicting mortality compared to previous parameters.

## Materials and methods

### Study population and design

**Fig. 1:**
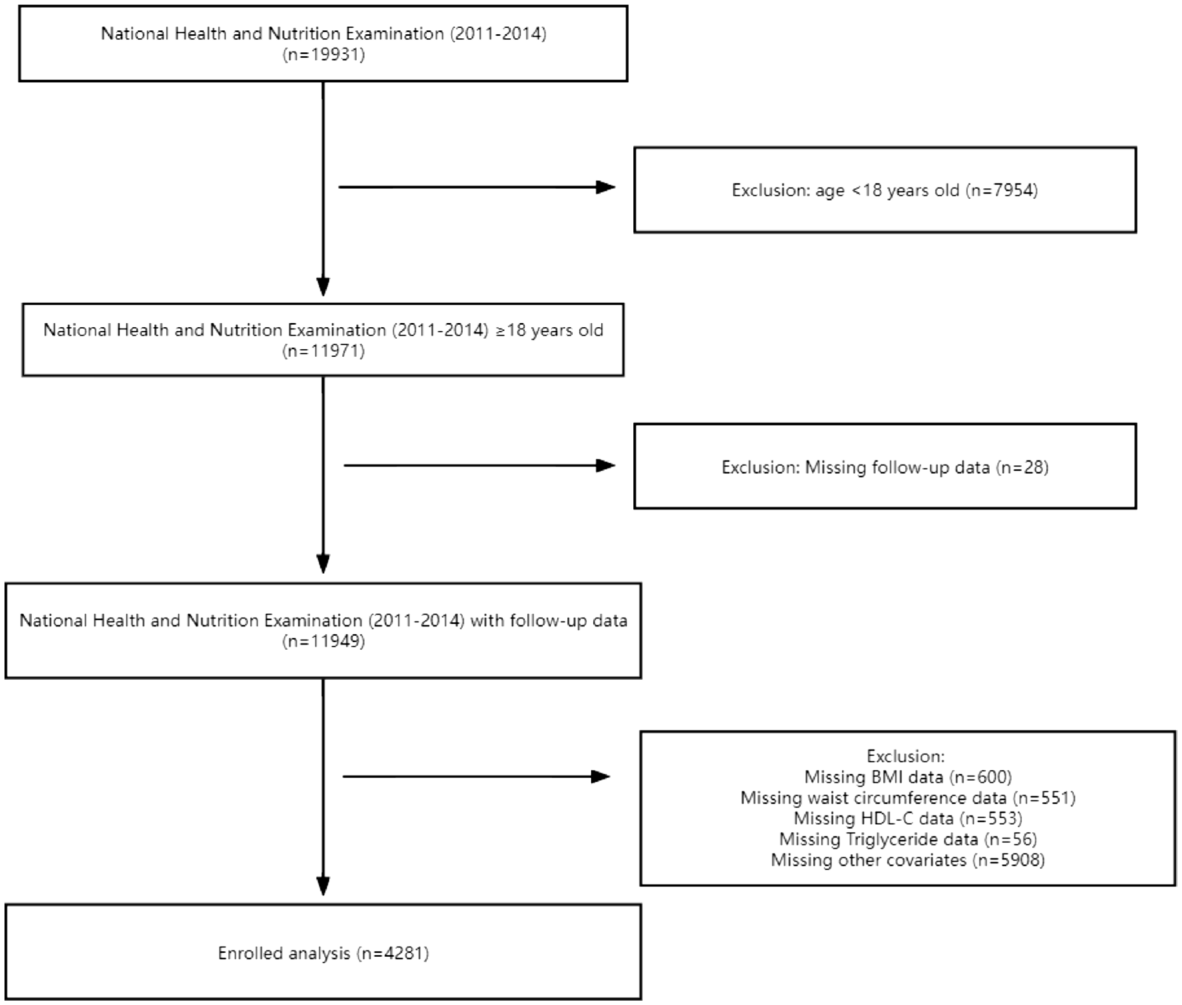
The research flowchart

The National Health and Nutrition Examination Survey (NHANES) is a cross-sectional study representing the health and nutritional status of the population within the USA. The Centers for Disease Control and Prevention has approved the NHANES protocol and written informed consent was obtained from all participants. This study enrolled 19,931 respondents from NHANES (2011–2014). A total of 7954 adults remained after excluding individuals less than 18 years. This study also excluded participants with missing VAI-related data (*n* = 1760) and other covariates (*n* = 5908) and individuals without follow-up data (*n* = 28). Finally, 4281 subjects were included. Informed consent was obtained from each subject (Fig. 1).

### Assessment of outcomes

We observe all-cause mortality as the end point. Participants were followed up through December 31, 2015 unless dropping out or meeting the intended outcome. The mortality status of participants was authenticated by matching with the National Death Index. Referring to the International Classification of Disease, 10th Edition (ICD-10), Clinical Modification System codes, cardiovascular mortality was identified as death caused by CVD or cerebrovascular disease (ICD-10 codes I00–I09, I11, I13, I20 to I51, and I60–I69).

### Anthropometric and serum biochemical parameters

Anthropometric and biochemical data were collected during the NHANES study. Waist circumference (WC) was measured at the iliac crest using a tape measure with a precision of 1 mm. Body mass index (BMI) was calculated by dividing weight in kilograms by the square of height in meters. A blood sample was collected from the antecubital vein of all participants by a trained phlebotomist. Detailed information regarding laboratory testing for triglycerides (TG), total cholesterol (TC), low-density lipoprotein cholesterol (LDL-C), high-density lipoprotein cholesterol (HDL-C), glycated hemoglobin (HbA1c), glucose and insulin levels can be found in the NHANES Laboratory/Medical Technician Procedures Manual [21, 22]. VAI was calculated as follows:

$$\mathrm{For males}:{\text{VAI}}= \left(\frac{{\text{WC}}}{39.68+1.88\times {\text{BMI}}}\right) \times \left(\frac{{\text{TG}}}{1.03}\right) \times \left(\frac{1.31}{{\text{HDL}}}\right);$$

$$\mathrm{For females}:{\text{VAI}}= \left(\frac{{\text{WC}}}{36.58+1.89\times {\text{BMI}}}\right) \times \left(\frac{{\text{TG}}}{0.81}\right) \times \left(\frac{1.52}{{\text{HDL}}}\right).$$

The multivariate regression model incorporated age, race, education level, marital status, hypertension, diabetes, smoking status, WC, BMI, HDL-C, TG, total cholesterol, and glucose. Age was significantly associated with mortality after adjusting for potential confounders and parameters included in VAI. The new index was defined as age-adjusted visceral adiposity index (AVAI) including age, BMI, WC, HDL-C, and TG. AVAI was estimated as follows:

$$\mathrm{For males}:-10.727+0.101\times {\text{age}}-0.108\times {\text{BMI}}-0.043\times {\text{WC}}-1.157\times {\text{HDL}}-{\text{C}}+0.075\times {\text{TG}};$$

$$\mathrm{For females}:-16.186+0.144\times {\text{age}}-0.013\times {\text{BMI}}+0.038\times {\text{WC}}-1.369\times {\text{HDL}}-{\text{C}}-0.151\times {\text{TG}},$$

where both TG and HDL levels are expressed in mmol/L[10].

### Covariates

The standardized questionnaires and physical examinations were used to assess covariates, including age, sex, race, education level, marital status, smoking status, alcohol consumption, history of hypertension, and diabetes. Disease states were defined in detail. Individuals with an average systolic blood pressure (SBP) ≥ 140 mmHg or an average diastolic blood pressure (DBP) ≥ 90 mmHg, or those diagnosed with hypertension and receiving antihypertensive medication, were classified as having hypertension. Participants with a fasting plasma glucose level ≥ 7.0 mmol/L or an HbA1c level ≥ 6.5%, as well as those with a diagnosed case of diabetes, were designated as having diabetes.

### Statistical analysis

All estimates were calculated accounting for NHANES sample weights. AVAI was expressed as quartiles. Continuous variables were checked for normal distribution before analysis, and these variables did not follow a normal distribution. Thus, continuous variables are presented as median (quantile 1st, quantile 3rd), while categorical variables are expressed as percentages, respectively. We conducted Kruskal–Wallis test (continuous variables) or Chi-square tests (categorical variables) to calculate the differences among different groups. Taking into account the complex survey design in NHANES, weighted Cox regression models were constructed to explore the association between AVAI and mortality risk instead of traditional Cox regression, which was expressed as calculated odds ratios (ORs) and 95% confidence interval (95% CIs). Crude analysis adjusted for no covariate (model 1), adjusted model adjusted for age, gender and race (model 2), fully adjusted model adjusted for age, gender, race, marital status, education level, smoking status, hypertension, diabetes, level of systolic blood pressure, glucose and total cholesterol (model 3). Kaplan–Meier survival analyses were applied to analyze the differences of survival rates according to AVAI groups, and the differences were examined by log-rank test. We also conducted smooth curve fittings to address the nonlinearity of AVAI and mortality after adjustment for the same covariates as in the Cox regression models. Then two-piecewise linear regression models were constructed to examine the difference of relationship at the threshold. The point with the highest likelihood among all the possible values was chosen as the threshold value. A logarithmic likelihood ratio test evaluated the differences between two-piecewise linear regression models. Receiver operator characteristic (ROC) curve was used to compare the predictive ability for mortality among AVAI and classical obesity-related parameters.

Empower stars (www.empowerstats.com, X&Y Solutions Inc., Boston, MA) was used for all statistical analyses. *P* < 0.05 was considered statistically significant.

## Results

### Population characteristics

This study included 4256 participants aged ≥ 18 years old from NHANES (2011–2014). Among all the participants, the medium age was 48 (34–63) years, and 49.87% were male. Based on AVAI quartiles, the basic characteristics are presented Table 1. All baseline covariates have significant differences among the AVAI groups (all *p* < 0.01). Subjects with higher AVAI scores had lower HDL-C concentrations and higher age, BMI, WC, glucose level, HbA1c, TG, and systolic blood pressure than those with lower VAI scores (*p* < 0.01). Meanwhile, individuals with higher AVAI scores were mostly likely to be male, smokers, and those with hypertension and diabetes. Throughout the 35 months of average follow-up period, 3.34% all-cause deaths and 1.05% cardiovascular deaths occurred.

**Table 1:** Basic characteristics of the population categorized by AVAI scores

AVAI	Q1	Q2	Q3	Q4	*p*-value ^1^
	≤ −8.36 (*n* = 1070)	− 8.36–6.51 (*n* = 1071)	− 6.51 to − 4.68 (*n* = 1071)	≥ −4.68 (*n* = 1069)	
Age (years)	28.00 (23.00–36.00)	40.00 (33.00–47.00)	54.00 (48.00–60.00)	71.00 (64.00–78.00)	< 0.001
Male, *n* (%)	252 (23.60%)	586 (54.66%)	637 (59.76%)	660 (61.40%)	< 0.001
Hypertension, *n* (%)	113 (10.58%)	317 (29.57%)	552 (51.78%)	786 (73.12%)	< 0.001
Diabetes, *n* (%)	21 (1.97%)	94 (8.77%)	220 (20.64%)	367 (34.14%)	< 0.001
Smoking, *n* (%)	329 (30.81%)	444 (41.42%)	500 (46.95%)	597 (55.59%)	< 0.001
Race, *n* (%)					< 0.001
Non-Hispanic White	391 (36.61%)	419 (39.09%)	408 (38.27%)	582 (54.14%)	
Non-Hispanic Black	217 (20.32%)	224 (20.90%)	254 (23.83%)	203 (18.88%)	
Mexican American	123 (11.52%)	171 (15.95%)	111 (10.41%)	94 (8.74%)	
Others	337 (31.55%)	258 (24.07%)	293 (27.49%)	196 (18.23%)	
Education level, *n* (%)					0.972
Under high school	154 (14.42%)	233 (21.74%)	253 (23.73%)	322 (29.95%)	
High school	195 (18.26%)	222 (20.71%)	247 (23.17%)	250 (23.26%)	
Over high school	719 (67.32%)	617 (57.56%)	566 (53.10%)	503 (46.79%)	
Marital status, *n* (%)					< 0.001
Married/cohabiting	538 (50.37%)	678 (63.25%)	686 (64.35%)	665 (61.86%)	
Widowed/divorced	91 (8.52%)	169 (15.76%)	251 (23.55%)	359 (33.40%)	
Never married	439 (41.10%)	225 (20.99%)	129 (12.10%)	51 (4.74%)	
BMI (kg/m^2^)	24.60 (21.70–29.00)	28.25 (24.60–32.50)	28.30 (24.83–32.80)	28.80 (25.65–33.25)	< 0.001
Waist circumference (cm)	85.20 (78.00–96.20)	96.40 (88.20–107.00)	100.00 (91.30–109.47)	104.20 (95.80–114.85)	< 0.001
SBP, (mmHg)	110.67 (104.00–118.00)	117.33 (109.33–126.67)	123.67 (114.67–135.33)	130.00 (119.33–141.67)	< 0.001
HbA1c (%)	5.20 (5.00–5.40)	5.40 (5.20–5.70)	5.70 (5.40–6.00)	5.90 (5.50–6.40)	< 0.001
Glucose (mmol/L)	5.16 (4.83–5.44)	5.44 (5.11–5.83)	5.66 (5.27–6.27)	6.00 (5.50–6.99)	< 0.001
HDL- C (mmol/L)	1.47 (1.24–1.76)	1.28 (1.06–1.55)	1.29 (1.09–1.60)	1.24 (1.06–1.45)	< 0.001
Triglyceride (mmol/L)	0.85 (0.61–1.18)	1.15 (0.81–1.72)	1.25 (0.87–1.92)	1.35 (0.95–1.99)	< 0.001
Total cholesterol (mmol/L)	4.55 (4.03–5.20)	5.02 (4.37–5.67)	5.17 (4.45–5.82)	4.66 (3.98–5.38)	< 0.001
LDL-C (mmol/L)	2.56 (2.10–3.08)	3.03 (2.46–3.65)	3.10 (2.53–3.70)	2.69 (2.10–3.32)	< 0.001
VAI	0.97 (0.62–1.59)	1.44 (0.88–2.36)	1.57 (0.92–2.71)	1.85 (1.14–2.93)	< 0.001
Outcomes, (%)					
All-cause mortality	4 (0.37%)	8 (0.75%)	23 (2.16%)	108 (10.05%)	< 0.001
Cardiovascular mortality	0 (0.00%)	0 (0.00%)	5 (0.47%)	40 (3.72%)	0.010

Data were expressed as *n* (%) and median (interquartile range)

*AVAI* age-adjusted visceral adiposity index, *BMI* body mass index, *SBP* systolic blood pressure, *HDL-C* high-density lipoprotein cholesterol, *LDL-C* low-density lipoprotein cholesterol, *VAI* visceral adiposity index

### Adjusted VAI improves predictive performance for mortality

**Fig. 2:**
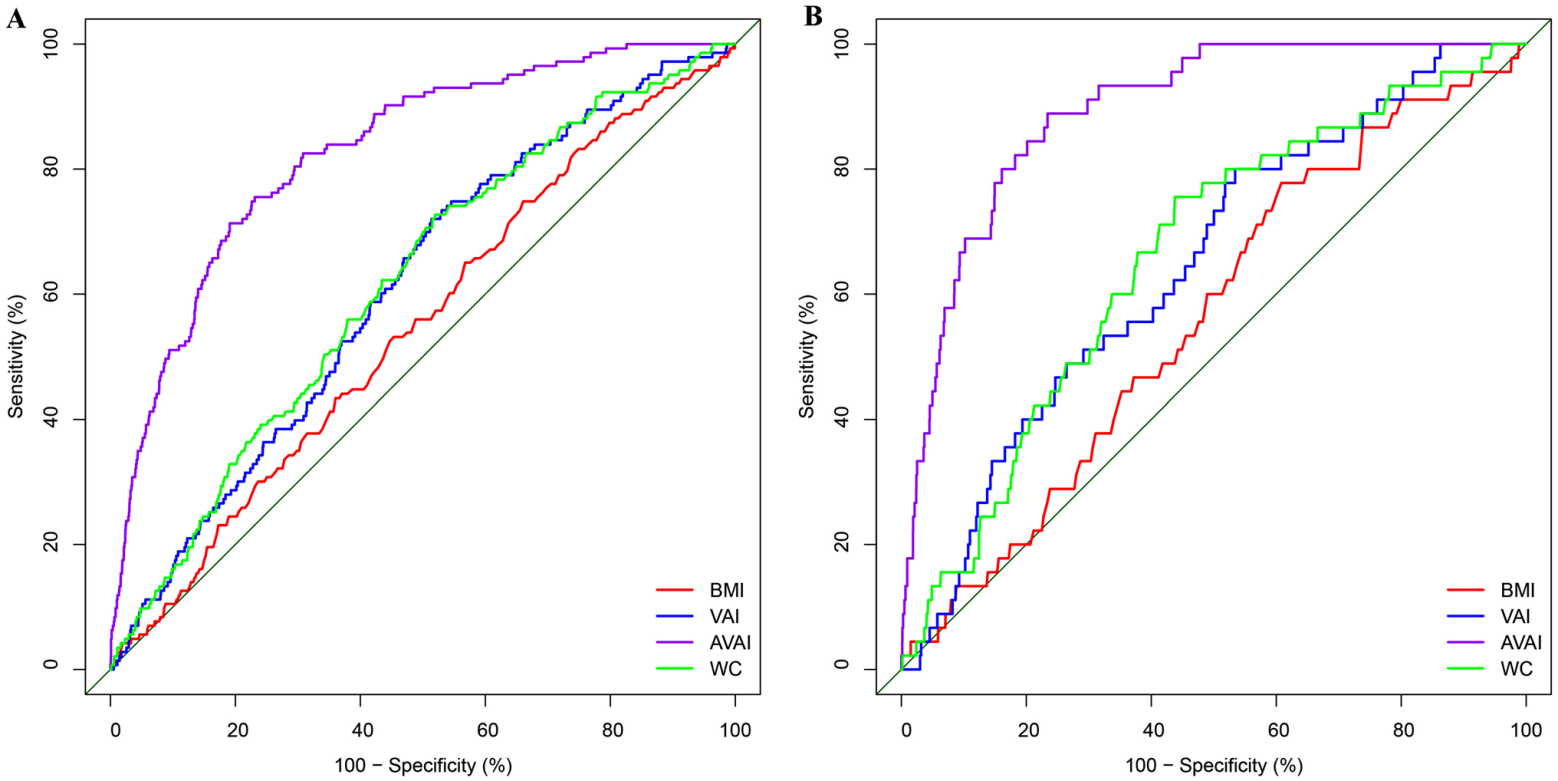
ROC analysis of all-cause (**A**) and cardiovascular (**B**) mortality. *AVAI* age-adjusted visceral adiposity index, *BMI* body mass index, *VAI* visceral adiposity index, *WC* waist circumference, *ROC* receiver operating characteristic

ROC curve analysis was used to verify whether VAI has a better predictive ability for mortality than previously established parameters. The area under the ROC curve (AUCs) of VAI for all-cause and cardiovascular mortality was 0.60 (0.56, 0.65) and 0.64 (0.57, 0.72), showing a better predictive ability than BMI, but a lower predictive ability than WC. Moreover, the AUCs of AVAI were 0.82 (0.79, 0.86) and 0.89 (0.85, 0.92) for predicting all-cause mortality and cardiovascular mortality, which were superior to BMI, WC and VAI (Fig. 2 and Table 2).

**Table 2:** Areas under ROC analysis of different parameters to predict cause-specific mortality

Variables	All-cause mortality, AUC (95%CI)	Cardiovascular mortality, AUC (95%CI)
BMI	0.54 (0.50, 0.59)	0.56 (0.48, 0.64)
WC	0.61 (0.57, 0.66)	0.66 (0.58, 0.73)
VAI	0.60 (0.56, 0.65)	0.64 (0.57, 0.72)
AVAI	0.82 (0.79, 0.86)	0.89 (0.85, 0.92)

*AVAI* age-adjusted visceral adiposity index, *BMI* body mass index, *VAI* visceral adiposity index, *WC* waist circumference, *ROC* receiver operating characteristic, *AUC* area under ROC curve, *CI* confidence interval

### The relationship of AVAI with mortality

**Fig. 3:**
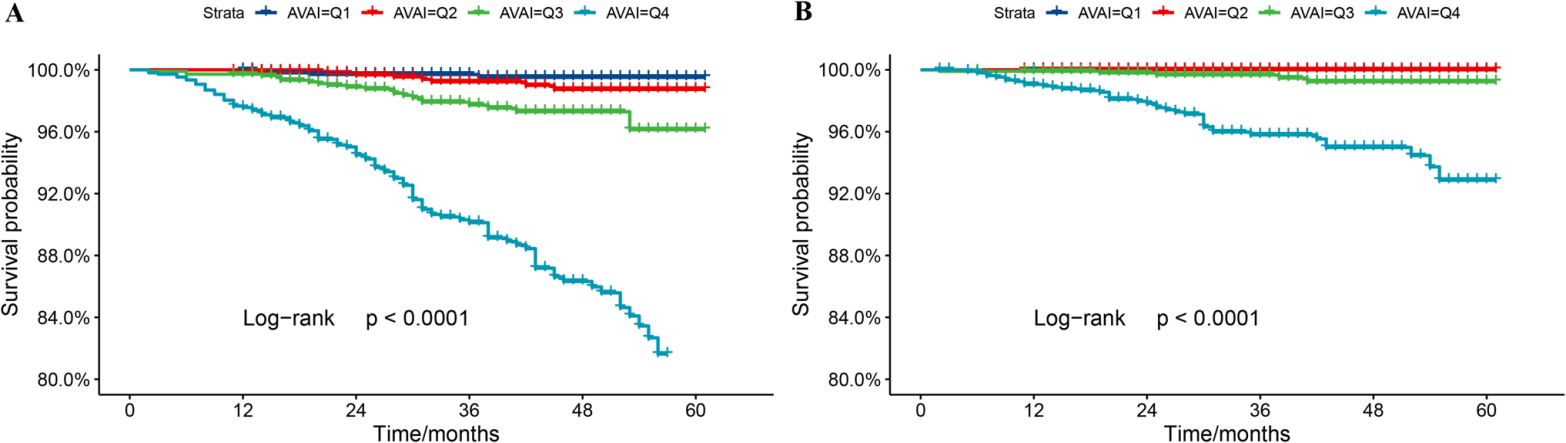
Kaplan–Meier survival curves for all-cause (**A**) and cardiovascular (**B**) mortality according to AVAI quartiles

Kaplan–Meier survival curves were diverged according to AVAI quartiles. The highest risk for all-cause and cardiovascular mortality was observed in the group with the highest AVAI score (log-rank *p* < 0.001) (Fig. 3). Weighted multivariate Cox regression was used to evaluate the relationship between AVAI and mortality risk. After adjusting for potential confounders, compared with the reference (Q1), the HRs (95% CI) for all-cause death was 1.13 (1.12, 1.14), 1.92 (1.90, 1.94), and 2.33 (2.30, 2.36) for Q2, Q3, and Q4 groups, respectively (all *P* for trend < 0.01). Weighted multivariate Cox regression failed to detect any relationship between AVAI and cardiovascular mortality due to inefficient cardiovascular events (Table 3).

**Table 3:** Multivariate Cox regression analysis of AVAI with all-cause mortality

	AVAI				*P* for trend
	Q1	Q2	Q3	Q4	
	≤ − 8.36	(− 8.36, − 6.51)	(− 6.51, − 4.68)	≥ − 4.68	
	HR (95%CI)	HR (95%CI)	HR (95%CI)	HR (95%CI)	
Median follow-up time (year)	3.00	2.91	2.91	2.75	
All-cause mortality					
Death, *n* (%)	4 (0.37%)	8 (0.74%)	23 (2.14%)	108 (10.10%)	
Death/1000 person-year	2.17	5.84	14.87	74.74	
Model 1	Reference	2.13 (2.11, 2.15)	8.07 (8.01, 8.14)	28.71 (28.48, 28.94)	< 0.01
Model 2	Reference	1.11 (1.10, 1.12)	2.14 (2.12, 2.16)	3.45 (3.41, 3.49)	< 0.01
Model 3	Reference	1.13 (1.12, 1.14)	1.92 (1.90, 1.94)	2.33 (2.30, 2.36)	< 0.01

Values are presented as weighted hazard ratios (HRs), 95% confidence interval (95%CI), and *P* value

Model 1 adjusted for none

Model 2 adjusted for age, gender, and race

Model 3 adjusted for age, gender, race, education level, marital level, smoking status, hypertension, diabetes, systolic blood pressure, glucose, and total cholesterol

### Dose–response relationship between AVAI and mortality risk

**Fig. 4:**
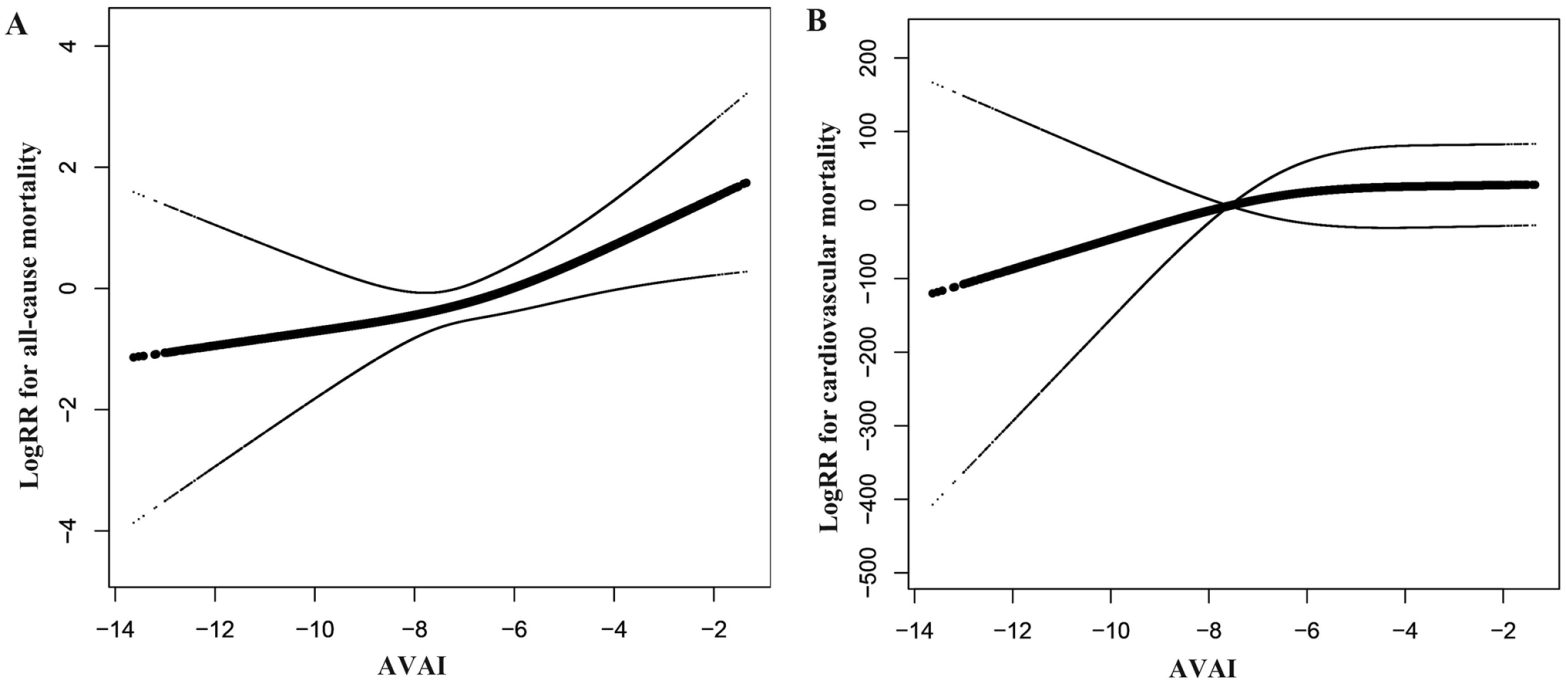
Relationship between AVAI and all-cause (**A**) and cardiovascular (**B**) mortality

We also tried to use smooth curve fittings to find the non-linear relationships between AVAI and mortality risks (Fig. 4). The results of standard logistic regression and two-piecewise linear regression are presented in Table 4. According to two-piecewise linear regression, the cutoff values were − 3.46 for all-cause mortality and − 4.59 for cardiovascular mortality, but the linear relationships were demonstrated by standard linear regression. Therefore, there is a dose–response relationship between AVAI and cause-specific mortality.

**Table 4:** Threshold and saturation effect analysis of AVAI on cause-specific mortality

Outcome	All-cause mortality HR (95% CIs) *P *value	Cardiovascular mortality HR (95% CIs) *P *value
Model1 Fitting model by standard linear regression	1.37 (1.03, 1.81) 0.028	3.60 (1.51, 8.59) 0.003
Model2 Fitting models by two-piecewise linear regression		
Inflection point	− 3.46	− 4.59
< Inflection point	1.24 (0.92, 1.68) 0.157	0.00 (0.00, 0.00) 0.886
> Inflection point	1.80 (1.14, 2.82) 0.010	2.81 (1.10, 7.19) 0.030
*P* for log-likelihood ratio test	0.143	0.054

The two-piecewise regression models were adjusted for age, gender, race, education level, marital level, smoking status, hypertension, diabetes, systolic blood pressure, glucose, and total cholesterol

*HR* hazard ratios, *95% CI* 95% confidence interval

## Discussion

This was a large-scale, multi-ethnic analysis evaluating the association between AVAI scores and all-cause and cardiovascular mortality in US adults. Individuals with higher AVAI scores also have more cardiovascular risk factors, such as hypertension, diabetes and hyperlipidemia. ROC demonstrated that AVAI is a better predictor than traditional obesity-related parameters for all-cause and cardiovascular mortality, which have higher sensitivity and specificity. Further Cox regression found that AVAI scores are independently associated with mortality in this study regardless of the socioeconomic, medical condition, lifestyle factors, and other covariates. The relationships between AVAI and cause-specific mortality are dose-responsive.

Visceral adiposity promotes metabolic dysfunctions, including type 2 diabetes, hypertension, and hyperlipidemia [23–27]. However, it is difficult to quantify visceral adipose tissues. VAI can be used to reflect visceral adiposity and adipose tissue dysfunction [10]. Some studies have found that VAI is significantly associated with diabetes, hypertension, and cardiovascular disease [28–36]. One study discovered that the VAI based on simple measurement has a better predicting ability for CVD than other simple ratios, e.g., BMI and WC [37], However, in the present study, VAI did not show better predicting performance for mortality than waist circumference. The inconsistency could be due to the differences in race and age among the studied cohorts. According to previous studies, aging is an important process influencing body fat distribution and mortality. Evidence has shown that a combination of metabolic syndrome parameters with age can enhance its predictive power [38]. We successfully demonstrated that age-adjusted VAI is independently associated with all-cause mortality and is a better indicator for mortality than simple anthropometric parameters and VAI.

However, obesity has been demonstrated to be a heterogeneous disorder, and there are differences between subcutaneous areas and visceral adipose tissue, including anatomical, cellular, molecular, and physiological aspects [39]. In contrast to subcutaneous adipose tissue, the adipocytes in VAT are larger and dysfunctional, and large adipocytes are insulin resistant and hyperlipolytic, resulting in a higher level of plasma glucose and free fatty acids (FFAs) [40]. Meanwhile, visceral adipose tissue is characterized by being rich in blood supply, and can be seen as an endocrine organ due to its secretory function [41, 42]. Visceral adipose tissue (VAT) adipocytes expand under excess energy intake, and synthesize a variety of proinflammatory proteins, termed adipokines, including leptin, monocyte chemoattractant proteins (MCP-1), tumor necrosis factor (TNF-a) and IL-6. These adipokines contribute to a chronic, low-grade inflammatory state in obesity, which raises the metabolic risk [6, 41–46]. Although VAI is a relatively comprehensive and easily measurable index for visceral adipose and significantly associated with insulin resistance, it does not take age into account and cannot comprehensively predict metabolic risk. Similar to obesity, aging is also an important factor influencing the function of visceral adipose tissue and plays a crucial role in many chronic diseases, including cancer, neurodegeneration, and CVD [47, 48]. Aging influences the function of visceral tissue through many mechanisms. Firstly, adipose tissue redistribution occurs with age, and the growth in fat mass occurs mostly with the expansion of the visceral adipose tissue due to the declining levels of hormones, such as testosterone, estrogen, and norepinephrine [49–51]. Removing VAT in rats through surgery could improve glucose tolerance, reduce liver triglycerides and increase life span [52, 53]. Secondly, aging also increases the size of VAT depot and promotes larger adipocytes in VAT, and further increases the expression of proinflammatory adipokines in hypertrophic adipocytes [54]. Based on in vitro experiments, older adipocytes decreased the gene expression of adiponectin and leptin and showed significant leptin resistance compared to young mature adipocytes [55]. Aging also processes the activation of stress response through the mitochondrial pathway, which increases reactive oxygen species [56]. Thirdly, immune cells are important component of VAT, such as adipose tissue macrophages (ATMs) [57]. Studies have demonstrated that the proportion of M2-like ATMs significantly decreases in aged mice, and aged ATMs showed increased secretion of proinflammatory cytokines and decreased expression of PPARγ [58, 59]. Therefore, aging is an important factor contributing to the inflammatory state. The term “inflammaging” was used to characterize the chronic low-grade inflammation triggered by endogenous signals that accompany aging. This inflammatory condition raises the incidence of cardiovascular disease, type 2 diabetes, and neurological disorders in geriatric patients [60–63]. Retrospective observational studies associate metformin with increased human life span and fewer age-related diseases by suppressing adipocyte proinflammatory responses [64]. Therefore, age is significantly associated with dysfunction of adipose and mortality and is essential for predicting mortality risk.

Based on the above discussion, we can conclude that AVAI has certain guiding significance, especially for geriatric patients. Firstly, geriatric patients often have insufficient awareness of changes in their body composition, making it difficult for them to adhere to the detection of such changes, especially with time-consuming and laborious examinations like CT scans. AVAI, as a simple indicator, can be calculated using routine biochemical and physical examination data. This is very helpful for geriatric patients to understand the changes in their body composition, particularly abdominal fat, and to manage it by changing dietary habits and increasing physical exercise. Secondly, AVAI shows good correlation with other metabolic indicators such as blood glucose, blood pressure, and blood lipids, reflecting the overall metabolic burden in geriatric individuals. AVAI can serve as an indicator for assessing the metabolic health status of geriatric patients. By monitoring changes in AVAI, doctors can intervene early and improve the metabolic health of geriatric patients. Lastly, our study found that AVAI outperforms other indicators in predicting mortality risk, which helps doctors assess the risk of mortality in geriatric patients and take appropriate intervention measures in a timely manner.

This study has some strengths, including the large nationwide, representative of US adults and adjustment for main covariates. However, the study also has some limitations. First, visceral adiposity was not measured directly. Second, in the study some variables like smoking status and medical history may lead to recall bias. Moreover, the findings are only applicable to the population in North America and should be cautiously applied to the population in other regions. Data from areas except the USA may help assess whether AVAI is also superior in other races.

## Conclusion

Age significantly improves the ability of VAI for predicting all-cause and cardiovascular mortality. Age-adjusted VAI is independently associated with mortality risk, and thus could be considered as a reliable parameter assessing mortality risk. We found that AVAI could stably predict all-cause and cardiovascular mortality in a dose-responsive manner.

## Data Availability

Publicly available datasets were analyzed in this study. This data can be found here: https://www.cdc.gov/nchs/nhanes/index.htm.
